# Low Magnitude of Compression Enhances Biosynthesis of Mesenchymal Stem Cells towards Nucleus Pulposus Cells via the TRPV4-Dependent Pathway

**DOI:** 10.1155/2018/7061898

**Published:** 2018-04-17

**Authors:** Yibo Gan, Bing Tu, Pei Li, Jixing Ye, Chen Zhao, Lei Luo, Chengmin Zhang, Zetong Zhang, Linyong Zhu, Qiang Zhou

**Affiliations:** ^1^National & Regional United Engineering Laboratory of Tissue Engineering, Department of Orthopedics, Southwest Hospital, Third Military Medical University (Army Medical University), 29 Gao Tan Yan Street, Shapingba District, Chongqing 400038, China; ^2^Institute of Rocket Force Medicine, State Key Laboratory of Trauma, Burns and Combined Injury, Third Military Medical University (Army Medical University), 30 Gao Tan Yan Street, Shapingba District, Chongqing 400038, China; ^3^Department of Orthopedic Surgery, No.89 Hospital of PLA, Weifang, 261026 Shandong, China; ^4^Key Laboratory for Advanced Materials, Institute of Fine Chemicals, East China University of Science and Technology, 130 Meilong Road, Shanghai 200237, China

## Abstract

Mesenchymal stem cell- (MSC-) based therapy is regarded as a promising tissue engineering strategy to achieve nucleus pulposus (NP) regeneration for the treatment of intervertebral disc degeneration (IDD). However, it is still a challenge to promote the biosynthesis of MSC to meet the requirement of NP regeneration. The purpose of this study was to optimize the compressive magnitude to enhance the extracellular matrix (ECM) deposition towards discogenesis of MSCs. Thus, we constructed a 3D culture model for MSCs to bear different magnitudes of compression for 7 days (5%, 10%, and 20% at the frequency of 1.0 Hz for 8 hours/day) using an intelligent and mechanically active bioreactor. Then, the underlying mechanotransduction mechanism of transient receptor potential vanilloid 4 (TRPV4) was further explored. The MSC-encapsulated hybrids were evaluated by Live/Dead staining, biochemical content assay, real-time PCR, Western blot, histological, and immunohistochemical analysis. The results showed that low-magnitude compression promoted anabolic response where high-magnitude compression induced the catabolic response for the 3D-cultured MSCs. The anabolic effect of low-magnitude compression could be inhibited by inhibiting TRPV4. Meanwhile, the activation of TRPV4 enhanced the biosynthesis analogous to low-magnitude compression. These findings demonstrate that low-magnitude compression promoted the anabolic response of ECM deposition towards discogenesis for the 3D-cultured MSCs and the TRPV4 channel plays a key role on mechanical signal transduction for low-magnitude compressive loading. Further understanding of this mechanism may provide insights into the development of new therapies for MSC-based NP regeneration.

## 1. Introduction

Intervertebral disc degeneration (IDD) has become a severe socioeconomic issue that attracts public attention in recent years. With the fastening of aging society, increasing number of people are influenced by lower back pain, neck pain, and reduced labor ability caused by IDD [1]. However, current treatments, including bed rest, exercise, physical therapy, and surgery, could only relieve the symptom but not remove the etiology of IDD, which cannot stop the development of IDD progression [2]. Therefore, there is an urgent need to find an effective treatment targeting the etiology of IDD.

Nucleus pulposus (NP), the central hydrated jelly-like tissue of the intervertebral disc (IVD), plays a core role in the process of IDD [3]. With the degeneration of the NP, the dehydrated NP lose its ability to exert the hydrostatic pressure within the IVD. Thus, the physiological tensile stress on outer annulus fibrosis (AF) is converted to the nonphysiological compressive stress. The change would accelerate the degeneration of AF and eventually lead to disc herniation. Therefore, repairing the degenerated NP is the key to the treatment of IDD. However, there is still no way to repair the NP because it lacks the capabilities of self-renewal and self-regeneration. Therefore, tissue engineering technology, particularly stem cell-based strategy to renew the NP, is the hot spot of current research for the treatment of IDD [4].

In recent years, research showed that mesenchymal stem cells (MSCs) have great potential in the field of NP regeneration [5]. MSC has shown several advantages as seed cells such as wide-range sources, easy accessibility, and ability of orientable differentiation, which makes it one of potential ideal cell source for NP regeneration. Previously, we successfully established a codelivery system of a dextran/gelatin hydrogel with TGF-*β*3-loaded PLGA nanoparticles that induced MSCs into discogenesis cells in situ [6]. Hiyama et al. recently reported that MSC-based tissue engineering strategy could be applied to repair the degenerated disc in canine [7]. These studies have shown the promising prospect to construct a tissue-engineered NP. A previous study has shown that a functional tissue-engineered NP needs to realize matrix deposition for encapsulated cells, which is the foundation of a repaired disc to bear mechanical loading [8]. However, the biosynthesis of MSC is relatively insufficient to meet the requirement even though we have proved that they could express the discogenesis phenotype with suitable orientation induction [6]. Thus, it is particularly important to find effective stimulation to enhance the biosynthesis of MSC.

Mechanical loading is one of the most effective external stimulations that significantly influence the cell fate [9]. Recent study has shown that the biosynthesis of the nucleus pulposus cells could be strengthened by proper magnitude of compressive loading when the disc was ex vivo cultured in a bioreactor [10]. Wang et al. also found that the expression of some critical genes which targets extracellular matrix (ECM) could be regulated with certain magnitude of compression [11]. However, whether could mechanical compression be applied to enhance the biosynthesis of MSC towards discogenesis is still indistinct. It is worth noting that the IVD is a load-bearing tissue *in vivo* and the mechanical stimulation is an ever-present external physical condition. If the proper magnitude of mechanical loading could be distinguished to boost the MSC biosynthesis, the function of the regenerative NP and subsequently the repaired IVD could be continuously restored using surgical technique.

In this study, the MSCs encapsulated in hydrogel were properly discogenesis induced with the previous 3D culture system. The biological effect of a relatively wide magnitude of the dynamic compression (5–20%) on encapsulated MSCs was studied using an intelligent and mechanically active bioreactor. The overall objective of this study was to investigate the potential of dynamic compression on the enhancement of the NP-like biosynthesis for MSCs. To achieve this, we first evaluated the cell viability of 3D-cultured MSCs under different magnitudes of dynamic compression. Second, we investigated the ECM deposition profile of 3D-cultured MSCs under the same mechanical environment. Finally, we further explored the underlying mechanism of its mechanical effect on the biosynthesis of MSCs.

## 2. Materials and Methods

### 2.1. Preparation of the TGF-*β*3-PLGANPs

The TGF-*β*3-loaded PLGANPs were prepared as previously described [6]. Briefly, 10 *μ*g of TGF-*β*3 (PeproTech, USA) was dissolved in 0.1 mL of distilled water. Thereafter, PLGA (50,50, Sigma-Aldrich, USA) was dissolved in dichloromethane (Sigma-Aldrich, USA). Later, the TGF-*β*3 was added to the PLGA solution and treated with ultrasonic in an ice bath. Then, the emulsion was added to 1% polyvinyl alcohol (PVA, Sigma-Aldrich, USA) aqueous solution to treat with ultrasonic. The obtained emulsion was transferred into PVA aqueous solution and stirred. Then, the solid particles were collected and washed with deionized water.

### 2.2. Isolation and Culture of Mouse MSCs

All the animal experiments followed the guidelines of the local Animal Ethics Committee (SYXK (YU) 2012-0012) of the Third Military Medical University. As a previous study [12], bone marrow-derived MSCs were collected from 6-week-old Balb/c mouse after euthanasia. The bone marrow was harvested by blushing the femurs and tibiae with the complete culture medium consisting of Dulbecco's modified Eagle's medium (DMEM, HyClone, USA), 10% fetal bovine serum (FBS, Gibco, USA), and 1% penicillin/streptomycin (Gibco, USA). Using density gradient centrifugation, the MSCs were isolated from the bone marrow and cultured in culture dishes (Corning, USA). The adherent cells were collected by trypsinization (0.05% trypsin-EDTA, Gibco, USA) after 5 days. The medium was changed every 3 days.

### 2.3. Establishment of the 3D Culture System for the MSCs

4A-PEG-acr (Mw 10,000, Jemkem Technology, China), oxidative dextran (Mw 100,000), and amino-modified gelatin were synthesized using previously described procedures [13]. The achieved solution was dialyzed by dialysis membrane (JingKeHongDa Biotechnology Co., China) in distilled water for 4 times per day to eliminate by-products. Finally, the mixture was lyophilized and cryopreserved. The IPN hydrogels were prepared as previously described [14]. Briefly, dextran, gelatin, and PEG solutions in PBS were mixed as the mass ratio of 5 : 3 : 2 to achieve a final polymer concentration of 10% while I2959 (Sigma-Aldrich, USA) concentration was 0.1%. Then, the P4 MSCs were premixed with precursor solutions at a density of 1 × 107 cells/mL. Then, the cell polymer mixture was injected into a cylindrical mold (*Ø* = 5 mm, height = 5 mm) for 1 min at 37°C to form the first network, followed by exposure to 365 nm UV light (5 W, UVATA, China) for 1 min to construct the second network. Then, the cell-encapsulated hydrogel was incubated in the culture dish in 5% CO_2_ at 37°C and the culture media was replaced once per day.

### 2.4. The Design of the Intelligent and Mechanically Active Bioreactor

**Figure 1:**
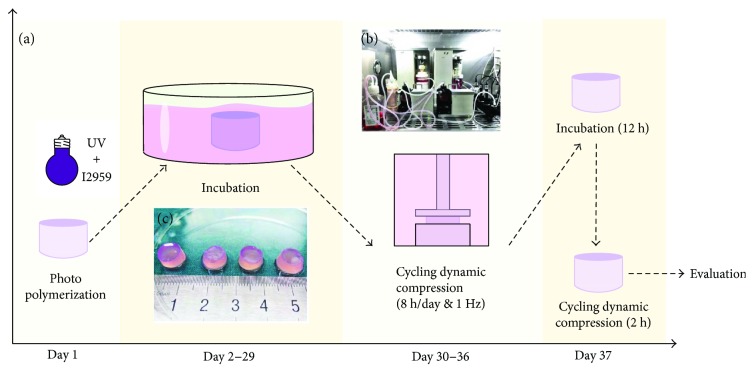
(a) Testing protocol of hybrid incubation and mechanical loading. The MSCs were encapsulated in the IPN hydrogel at day 1. The MSC-seeded hybrid was discogenesis induced and incubated for 28 days. Then, the composite hydrogel was treated with cyclic compressive loading for 7 days (5%, 10%, and 20% at the frequency of 1.0 Hz for 8 hours) and then evaluated at day 36. (b) Overview image of the bioreactor platform and primary units of the bioreactor. (c) Gross observation of the MSC-encapsulated hydrogels after compressive loading.

The custom-built bioreactor mainly consists of a mechanical loading element, culture chamber, circulating perfusion equipment, biochemical composition monitoring platform, nutrient exchange equipment, and other attachments (Figure 1(b)). Mechanical loading is axially applied with an integrated servomotor integrated within the culture chamber. The magnitude of compression could be adjusted in real time based on feedback from a central controller. More details about the bioreactor could refer to our previous study [15].

### 2.5. Mechanical Loading Profile

Mechanical loading profile were illustrated in Figure 1(a). After 28 days of incubation, the cell-seeded hydrogels were transferred to the mechanically active bioreactor to conduct compressive loading. The hydrogels were randomly compressed with different magnitudes (5%, 10%, and 20%) at the frequency of 1 Hz for 8 hours per day. The compression durations (8 h) were chosen because it is the human physiological condition. The free swelling hydrogels were used as control. The bioreactor was perfused with fresh complete culture media at a rate of 15 mL/min for promoting nutrition supply and subsequently tissue remodeling. After 7 days of dynamic compression, the loaded hydrogels (Figure 1(c)) were transferred to the culture dishes to relax for 12 hours, followed by mechanical loading for 2 hours before sample evaluation.

To regulate TRPV4 channel activity under dynamic compression, media was supplemented with 10 *μ*M GSK205, a TRPV4-selective antagonist or 1 nM GSK101, a TRPV4-selective agonist (Sigma-Aldrich, USA), during the compression stage as the experimental groups. The control group received the same amount of vehicle (0.1–0.2% DMSO) at the same time. During the static culture, all the samples were treated with fresh media without the TRPV4 antagonist or agonist.

### 2.6. Live/Dead Assay

The cell viability with different magnitudes of compression was investigated with the LIVE/DEAD Viability Assay Kit (Invitrogen, USA), according to the manufacturer's instructions. Briefly, the hybrids were washed with PBS for 3 times and incubated with 1 mL of PBS containing 4 mM EthD-1 and 2 mM calcein AM for 30 min at 37°C (*n* = 3). Then, the washed hybrids were visualized by a laser scanning confocal microscopy (LSCM. Zeiss 780, Germany). Living cell/dead cell ratio was calculated by ImageJ software (Wayne Rasband, National Institute of Health, USA).

### 2.7. Biochemistry Assays

MSC hydrogel hybrids in each group were lyophilized (*n* = 3). Cellular DNA content, cell-associated glycosaminoglycan (GAG), and hydroxyproline (HYP) were measured. Double-stranded DNA content was determined via a Quant-iT™ PicoGreen® assay kit (Invitrogen, USA) in accordance with manufacturer's protocol. Total double-stranded DNA content in each sample was determined from a standard curve developed from serial dilutions of DNA stock provided. Total sulfated GAG content was quantified using the dimethylmethylene blue assay as described before [16]. GAG content was determined via a standard curve developed from serial dilutions of known concentrations of chondroitin sulfate sodium salt. HYP content was quantified according to methods adapted from the study of Blumenkrantz and Asboe-Hansen [17]. HYP content was determined using a standard curve of known concentrations of HYP.

### 2.8. Histological Analysis

The collected specimens (*n* = 3) were rinsed twice with PBS and fixed in 4% paraformaldehyde for 24 h. Then, they were then embedded in paraffin and sectioned in 4 *μ*m thickness. Serial sections were stained with Masson trichrome.

### 2.9. Immunohistochemical Analysis

For visualizing analysis of ECM deposition, collected hybrids were moved into paraffin and sectioned in 4 *μ*m thickness (*n* = 3). Thereafter, the sections were immunostained with aggrecan following a standard immunohistochemistry staining procedure. The aggrecan (1 : 400, sc-16492, Santa Cruz Biotechnology, USA) and goat anti-mouse horseradish peroxidase-conjugated secondary antibody (1 : 1000, CW0102, Cwbiotech, USA) were applied in the analysis.

### 2.10. Real-Time PCR Assay

To determine the sensing and responding pattern of the MSCs under different magnitudes of compression, the specific gene expressions of discogenesis including aggrecan, type II collagen, and Sox-9 were assessed using real-time polymerase chain reaction (RT-PCR) and GAPDH was used as the internal reference. The MSC hydrogel-seeded hybrids were treated with TRIzol (Geno Technology Inc., USA) and fully grounded. RNA was extracted and cDNA was generated by applying cDNA reverse transcription kit (Life Technologies, USA) and diluted to 5 ng/*μ*L. Gene expression was analyzed by quantitative RT-PCR (Applied Biosystems 7500, Thermo Fisher Scientific, USA). Data were calculated by the 2^−ΔΔCt^ method (*n* = 3). The primers used in this study are shown in Table 1.

### 2.11. Western Blotting Analysis

A semiquantitative analysis of differentiation was performed on cell-seeded hydrogels via Western blotting as described previously using the following antibodies that were diluted in 3% BSA in TBST buffer: aggrecan (1 : 1000, sc-16492, Santa Cruz Biotechnology, USA), Col II (1 : 5000, ab34712, Abcam, USA), and Sox-9 (1 : 1000, sc-20095, Santa Cruz Biotechnology, USA) antibodies and goat anti-mouse horseradish peroxidase-conjugated secondary antibody (1 : 2000, CW0102, Cwbiotech). And the protein level was quantified and normalized to GAPDH bands by densitometry in Quantity One software (version 4.6.2, Bio-Rad, *n* = 3).

### 2.12. Statistical Analysis

All of the quantitative data are presented as the mean ± standard deviation. One-way ANOVA was used to assess the statistical significance of results between groups by SPSS software (version 15.0, IBM, USA). Differences were considered significant when *p* < 0.05.

## 3. Results

### 3.1. Cell Viability of Encapsulated MSCs in the IPN Hydrogel under Dynamic Compression

**Figure 2:**
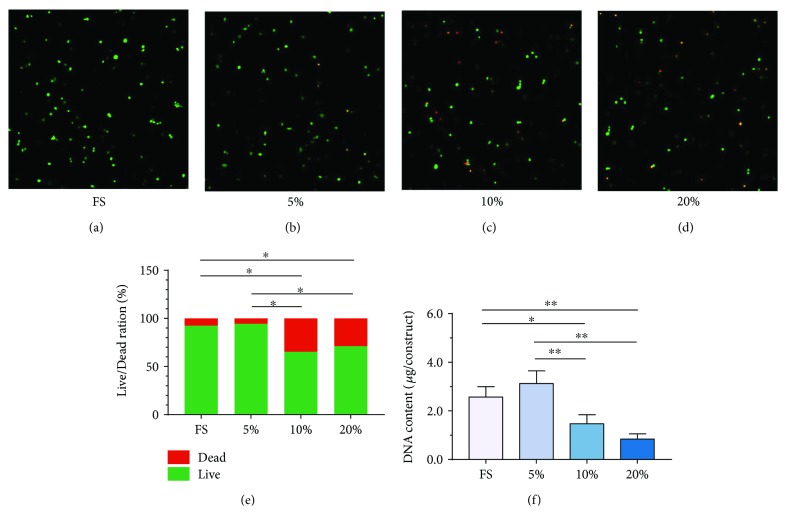
Cell viability of encapsulated MSCs in the IPN hydrogel. (a–d) A fluorescent Live/Dead staining for the MSC-encapsulated hybrids treated with free swelling (FS), compressive loading at magnitude of 5% (5%), compressive loading at magnitude of 10% (10%), and compressive loading at magnitude of 20% (20%) (magnification: 40x). (e) Quantification of NPC percent (Live/Dead) for the fluorescent Live/Dead staining. (f) Measurement of the DNA content of MSC-seeded hybrids treated with free swelling and compressive loading at magnitudes of 5%, 10%, and 20%. Data were expressed as means ± SD (*n* = 3). ^∗^*p* < 0.05 and ^∗∗^*p* < 0.01.

A fluorescent Live/Dead staining was used to visualize the cell viability within the hydrogels. Living cells are stained by calcium AM, which yields a green fluorescence. Membranes of dead cells comprise EthD-1, yielding a red fluorescence. Fluorescent images of MSC hydrogel hybrids were obtained after mechanical exposure (Figures 2(a)–2(d)). In the hybrids copied with no compression (free swelling (FS) group) or lower magnitude of compression (5% magnitude of compression (5%) group), the retentive cells were almost living and very few dead cells could be found. However, in the hybrids treated with moderate compression (10% magnitude of compression (10%) group) or high compression (20% magnitude of compression (20%) group), much more dead cells were presented although the living cells still accounted for the majority. Quantitative analysis of the data (Figure 2(e)) showed that the proportion of viable cells in the FS or 5% groups was significantly higher than those in the 10% or 20% groups (*p* < 0.05). Interestingly, there is no significant difference between the FS and 5% groups (*p* > 0.05). The data from DNA content analysis were in good agreement with the Live/Dead assay (Figure 2(f)). The results demonstrated that our 3D culture system exhibited good cytocompatibility and cell viability of MSCs under the absence of stress or low-magnitude compressive stress. In contrast, the viability of MSCs was declined when treated with moderate- or high-magnitude compressive stress. Low-magnitude mechanical environment is possible to be favorable to the compressed MSCs.

### 3.2. ECM Deposition under Different Magnitudes of Compressive Stress

**Figure 3:**
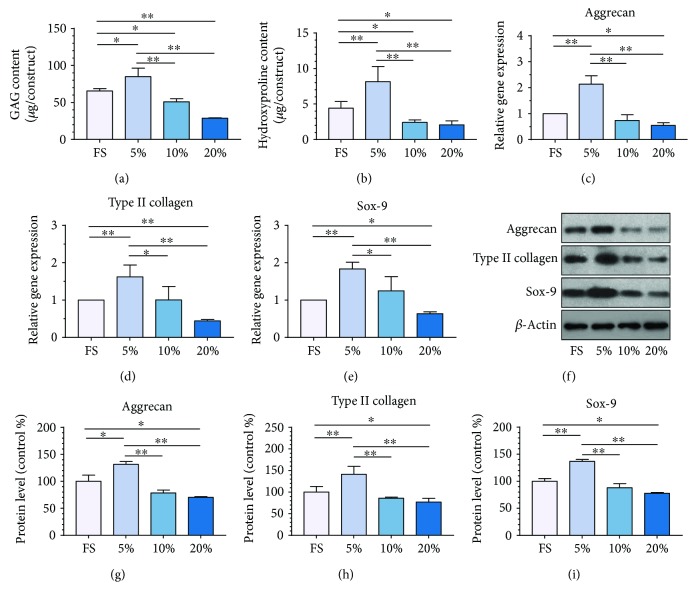
ECM deposition under different magnitudes of compressive stress. Measurement of the biochemical compositions, including (a) GAG content and (b) HYP content under different magnitudes of compressive stress. (c–e) The gene expressions of (c) aggrecan, (d) type II collagen, and (e) Sox-9 of MSC seeded in the hydrogel treated with free swelling and compressive loading at magnitudes of 5%, 10%, and 20%. The expression levels, quantified using real-time PCR, are normalized to those of housekeeping gene, GADPH. (f) Western blotting analysis of ECM proteins, aggrecan, type II collagen, and Sox-9 produced by MSCs cultured in the hydrogels treated with free swelling and compressive loading at magnitudes of 5%, 10%, and 20%. (g–i) The histogram of quantitative results of Western blot analysis. Data were normalized to the data obtained from MSCs cultured in the hydrogel treated with free swelling and evaluated on a relative basis for comparison between different samples. Data were expressed as means ± SD (*n* = 3). ^∗^*p* < 0.05 and ^∗∗^*p* < 0.01.

In order to study the effect of different magnitudes of compressive stress on the biosynthesis of MSCs encapsulated in the hydrogels, the ECM deposition was quantitatively analyzed by biochemical composition assay, RT-PCR, and Western blot. As shown in Figures 3(a) and 3(b), MSCs treated with low-magnitude compression exhibited significantly higher GAG (*p* < 0.05) and HYP (*p* < 0.01) contents compared with those of the FS group, demonstrating elevated ECM deposition under low-magnitude compressive stimulation. However, the biochemical composition under 10% or 20% magnitude of compression was significantly decreased compared with that in the FS group (*p* < 0.05). In the gene transcription level, the discogenesis ECM-related genes, including aggrecan, type II collagen, and Sox-9, were also significantly upregulated after 7 days of 5% magnitude of compression when compared with all the other three groups (Figures 3(c)–3(e)). With increasing magnitude of compression, the gene expression exhibited an obviously declining trend and the 20% group constantly showed the lowest gene expression among all the groups. At the protein level, the results were similar to the gene expression (Figures 3(f)–3(i)). The encapsulated MSCs presented the highest expression of ECM-related proteins when treated with low magnitude of compression whereas the lowest expression suffered from the high-magnitude of compression.

**Figure 4:**
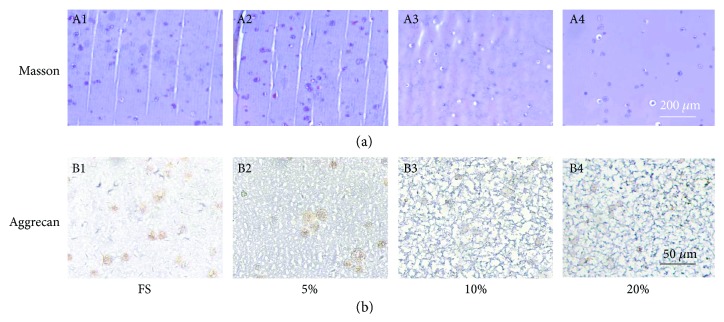
Histological observation of the MSC-encapsulated hybrids. (a) Photomicrographs showed Masson trichrome staining of the MSC-encapsulated hybrids treated with free swelling and compressive loading at magnitudes of 5%, 10%, and 20%. (b) The immunohistochemistry staining of the deposition of aggrecan in the MSC-encapsulated hybrids treated with free swelling and compressive loading at magnitudes of 5%, 10%, and 20%. White bar = 200 mm. Black bar = 50 mm. (*n* = 3).

The effect was further validated by histological and immunohistochemical staining (Figure 4). In the Masson trichrome staining, the chondrogenesis collagen fiber could be blue stained. As shown in Figure 4(a), the number and intensity of blue staining MSCs in the hydrogels were also much higher in the groups of FS and 5%. With the increasing of compressive magnitude, the blue staining cell number and intensity were decreasing gradually.

The immunohistochemistry for aggrecan showed the similar situation that low-magnitude compression strongly promoted aggrecan deposition in the encapsulated MSCs. All these data demonstrated that the biosynthesis of MSCs towards nucleus pulposus appeared as an obvious dose-response relationship, namely, low-intensity compression which promoted the anabolism, while high-intensity compression enhanced the catabolism.

### 3.3. The Anabolic Effect of Low-Magnitude Compression Is Inhibited by Inhibiting the Transient Receptor Potential Vanilloid 4 (TRPV4) Channel

**Figure 5:**
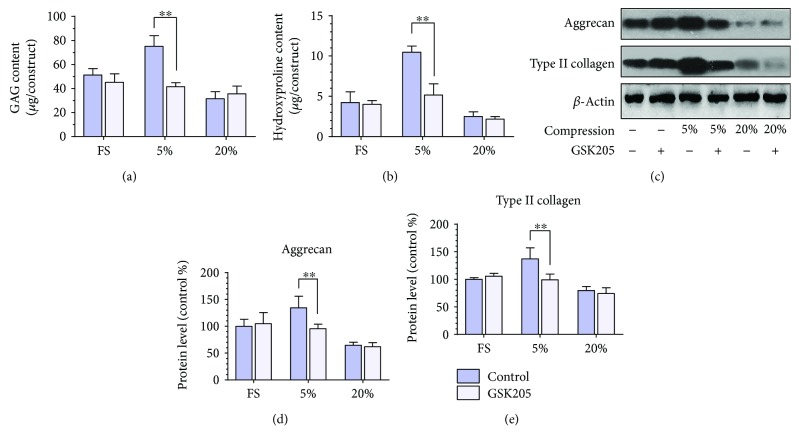
The anabolic effect of low-magnitude compression is inhibited by inhibiting the TRPV4 channel. Measurement of the biochemical compositions, including (a) GAG content and (b) HYP content treated with GSK205, the TRPV4 antagonists, during different magnitudes of compressive stress. The hybrids that were not treated with GSK205 were used as control. (c) Western blot analysis of ECM proteins aggrecan and type II collagen produced by MSCs cultured in the hydrogels treated with GSK205 during free swelling and compressive loading at magnitudes of 5%, 10%, and 20%. The hybrids that were not treated with GSK205 were used as control. (d–e) The histogram of quantitative results of Western blot analysis. Data were normalized to the data obtained from MSCs cultured in the hydrogel only treated with free swelling and evaluated on a relative basis for comparison between different samples. Data were expressed as means ± SD (*n* = 3). ^∗∗^*p* < 0.01.

TRPV4 has been recently reported to play an important role in the mechanosensitivity of chondrocyte [18]. We further investigated whether the function of TRPV4 is related to the mechanotransduction of 3D-cultured MSCs. The cell-seeded hybrids were dynamically loaded for 7 days with low- or high-magnitude compression, both in the presence and absence of GSK205, the TRPV4 antagonists. As shown in Figures 5(a) and 5(b), the GSK205 exposure alone had no effect on the GAG and HYP content in the FS group (*p* > 0.05). When treated with low magnitude of compression, the loading-induced elevated GAG and HYP content were partially attenuated by GSK205 (*p* < 0.01). However, GSK205 had no effect on the biochemical composition of the hybrids under high-magnitude compression (*p* > 0.05). The inhibiting effect of GSK205 was further validated in protein level by Western blot analysis. The results showed that GSK205 could significantly inhibit the anabolic effect of low-magnitude compression on the expressions of aggrecan and type II collagen (*p* < 0.05). Nevertheless, the ECM-related protein expressions were not affected by GSK205 in the FS and 20% groups (*p* > 0.05), which was consistent with the above data. These results demonstrated that the anabolism of 3D-cultured MSCs induced by low-magnitude compression was possible via a TRPV4 channel-dependent manner.

### 3.4. Activation of TRPV4 Enhanced the Biosynthesis Analogous to Low-Magnitude Compression

**Figure 6:**
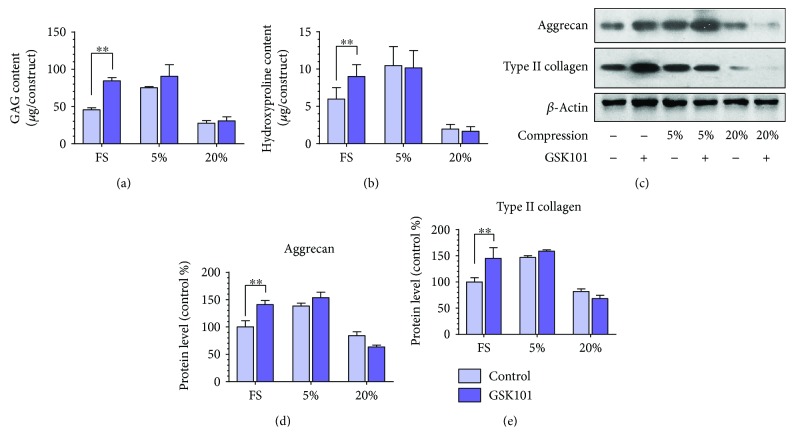
Activation of TRPV4 enhanced biosynthesis analogous to low-magnitude compression. Measurement of the biochemical compositions, including (a) GAG content and (b) HYP content treated with GSK101, the TRPV4 agonist, during different magnitudes of compressive stress. The hybrids that were not treated with GSK101 were used as control. (c) Western blot analysis of ECM proteins aggrecan and type II collagen produced by MSCs cultured in the hydrogels treated with GSK101 during free swelling and compressive loading at magnitude of 5%, 10%, and 20%. The hybrids that were not treated with GSK101 were used as control. (d–e) The histogram of quantitative results of Western blot analysis. Data were normalized to the data obtained from MSCs cultured in the hydrogel only treated with free swelling and evaluated on a relative basis for comparison between different samples. Data were expressed as means ± SD (*n* = 3). ^∗∗^*p* < 0.01.

In order to explore the function of the TRPV4 channel on the biosynthesis of MSCs, the MSC-encapsulated hybrids were exposed to the TRPV4 agonist GSK1016790A (GSK101) during the dynamic loading. Interestingly, as shown in Figures 6(a) and 6(b), GSK101 could significantly enhance the GAG and HYP syntheses in the hybrids in the FS group (*p* < 0.05). In contrast, the anabolic effect was not obvious when treated with dynamic loading, under neither the low magnitude of compression nor high magnitude of compression (*p* > 0.05). The result of the protein level is in good agreement with above results. The expressions of aggrecan and type II collagen are significantly upregulated by GSK101 (*p* < 0.01), and the anabolic effect of activating TRPV4 was similar to that of low-magnitude compression. GSK101 could slightly upregulate the ECM-related protein expression under low-magnitude compression but the effect was not significant (*p* > 0.05). No obvious effect of GSK101 on the protein expression could be found for the hybrids under high-magnitude compression. The above results illustrated that GSK101 processes a similar proanabolic function to low-magnitude compression by activating the TRPV4 channel.

## 4. Discussion

The therapy of IDD has drawn increasing attention owing to the rapidly growing number of patients with lower back pain or neck pain in recent years. Cell-based therapeutic strategy, particularly using MSC as cell source, shows promise in overcoming the self-renewal disability of NP cells, which was regarded as the difficulty to regenerate the degenerated IVD. To promote the efficiency of discogenesis for MSCs, it is critical to develop effective stimulation in inducing oriental differentiation and biosynthesis. The aim of the study is to optimize compressive magnitude to enhance the ECM deposition of the MSCs. Therefore, we investigated the biosynthesis effect of different magnitudes of compressive stress on a tissue-engineered NP developed by MSCs and explored the related mechanism preliminarily.

In the study, we constructed the 3D culture system for MSCs by using an IPN hydrogel as scaffold. Our previous study has shown that the IPN hydrogel is tough enough to resist the extremely high magnitude of compression without structural failure [14]. Meanwhile, it exhibited as friendly to the encapsulated cell for long-term culture. The advantages of mechanical strength and bioactivity make the IPN hydrogel a suitable 3D culture scaffold to conduct the mechanical biological study. It is as biomimetic as a previous ex vivo organ model [10] while as uncomplicated as a previous single-layer 2D cell model [19]. The annulus fibrosus would be injured inevitably when hydrogel was injected into the NP cavity. Previously, our study has showed the good injectability of IPN hydrogel and an illuminating device for minimal invasion was prepared. Thus, the IPN hydrogel-based 3D culture system is suitable to be used as cell carrier for disc regeneration *in vivo.* In addition, the mechanically active bioreactor could realize the dynamic mechanical loading which is closer to the actual situation of the IVD *in vivo* than the traditional hydrostatic pressure model [15]. Simultaneously, the monitoring of the magnitude of compression and nutrition compositions in the bioreactor decreases the error caused by confound factors. All these conditions make our model a suitable model for mechanical biological study.

IVD *in vivo* is subject to dynamic compression during daily activities. The persistent mechanical environment is extremely important for the biological behavior of the NP [20] including the regenerated NP using cell-based therapy. If the biological effect of different mechanical environments could be understood thoroughly, the mechanical environment could be utilized to alter the biological performance of the implanted tissue-engineered NP. A previous study indicates that the biological responses of disc cells to dynamic compression are magnitude dependent in disc cell culture and animal *in vivo* studies [21]. In the present study, low-magnitude compression had little effect on the cell viability compared with free swelling. However, the cell viability was decreasing rapidly with improving the compressive magnitude. This phenomenon was in agreement with our recent study which indicated that high-magnitude compression could increase the apoptotic cells within the immature NP [10].

IVD is a load-bearing organ essentially, and NP is the central component of the mechanical behavior in the IVD. Aggrecan, one of the major ECM compositions of a NP, could absorb the water into the NP and keep the balance of external loading and internal hydrostatic pressure of the NP, which is the determinant of the load-bearing ability of the NP [22]. Thus, the ECM synthesis and secretion are core functions of natural NP cells and the key parameter of a tissue-engineered NP. Obviously, low-magnitude compression (5%) could improve the expression of aggrecan and type II collagen of the MSC-encapsulated hybrids in mRNA and protein level compared with the free swelling hybrid. Sox-9, the key regulator of ECM synthesis towards chondrogenesis [23], was also significantly elevated by low-magnitude compression. In theory, low-magnitude compression is beneficial to the cell status because the persistent fluctuations of compression and relaxing lead to a pumping effect of the fluid exchange of the disc matrix [24]. The hydrogel scaffold is also a matrix full of retentive water. The phenomenon in this study should attribute to the dynamic compression could make the culture media movement into and out of the disc matrix, which is supportive to improve cell biological function as previous study [25]. In the study, low-magnitude compression could not influence the cell viability yet, but the biosynthesis was effectively changed. However, it is noteworthy that the high magnitude of compression had a negative effect on the biosynthesis of 3D-cultured MSCs, which is consistent with the pathology of IDD [26]. Our previous study also reported that a relatively high compressive magnitude can decrease matrix deposition within the immature NP [10].

**Figure 7:**
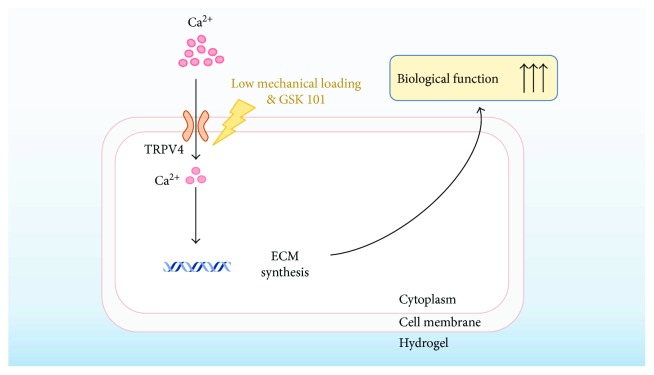
Schematic diagram. The low magnitude of compression and GSK101 could activate the TRPV4 channel on the cell membrane in MSCs. TRPV4, the Ca^2+^-preferred membrane ion channel, is possible to transduce the external mechanical signals into internal response of biosynthesis expression via the generation of intracellular Ca^2+^ influx. The gene expression could result in ECM synthesis and secretion at last, improving the biosynthesis capability of MSCs towards discogenesis.

TRPV4 is a Ca^2+^-preferred membrane ion channel which extensively participated in transducing external physical and chemical signals into internal biological responses via the generation of intracellular Ca^2+^ transients [27, 28]. O'Conor et al. recently reported the TRPV4-dependent anabolic response of chondrocytes to the mechanical stimulation, suggesting that TRPV4 plays a key role in the maintenance of matrix homeostasis under mechanical loading [18]. The upregulated biosynthesis under the low-magnitude of compression in MSCs made us hypothesize that TRPV4 might be implicated in the process of mechanotransduction. In the study, the inhibition of TRPV4 made mechanically induced elevated ECM biosynthesis and matrix accumulation totally compromised. The surprising result indicated that TRPV4 plays a critical role in transducing mechanical signals of MSCs, especially for low-magnitude compression. We also found that the activation of TRPV4 by a chemical agonist could induce analogous anabolic effect on ECM with the low magnitude of compression, further confirming that these anabolic responses of MSCs were transduced via TRPV4. It has been reported that Ca^2+^ transient signaling is one of the earliest events in response to mechanical loading [29, 30]. In the process of low-magnitude compression on the MSC-encapsulated hybrids, TRPV4 could lead the Ca^2+^ influx and subsequently transduce a diverse set of biosynthesis responses (Figure 7). However, it is confusing why the ECM expressions under the high magnitude of compression were not influenced by TRPV4. Maybe, the dominant mechanotransduction mechanism is different for MSCs under different magnitudes of compression. The mechanism of the TRPV4 channel during MSC compressive stress remains to be studied further.

Previously, researchers had used growth factors or phytochemicals to enhance ECM synthesis [30–32]. However, the establishment of a long-term controlled release system is the hindrance for these drugs' application [33]. Toh et al. also reported that the relative softer scaffold with lower crosslinking density could also promote matrix deposition of MSCs towards chondrogenesis [34]. But the rapid degradation of soft hydrogel with low crosslink density might influence the effect in long term. We focused on mechanical loading owing to its long-term effect. A recent study has shown that the dynamic fixation system for treatment of IDD could reduce the mechanical bearing of the target disc [35]. What is more, the degenerated disc could be rehydrated after reducing the loading by the kind of treatment in some cases [36]. It could be a promising strategy to combine implantation with MSC-based hydrogel and fixation with the precise mechanical control system to make the NP regeneration into reality. There are some limitations in this study. The biological response of murine MSCs to mechanical loading is probably different from that of *Homo sapiens* although a recent study has shown similar effects under mechanical loading [37]. Another limitation was that the status of MSCs *in vitro* is possible to be different from those *in vivo* because the microenvironment of the NP *in vivo* is of high acidity and low nutrient supply. In the future, an *in vivo* study should be conducted to explore the mechanical biological mechanism of MSCs by hydrogel injection in upright animals.

## 5. Conclusion

In summary, in this study, we investigated the ECM biosynthesis of a MSC-encapsulated hydrogel 3D culture system under different magnitudes of compression using an intelligent and mechanically active bioreactor. The findings from this study demonstrate that the biosynthesis of MSCs to dynamic compression is magnitude dependent. Low-magnitude compression promoted the anabolic response of ECM deposition towards discogenesis where high-magnitude compression induced the catabolic response for the 3D-cultured MSCs. The study further revealed the profound effects of mechanotransduction pathways that TRPV4 channel plays a key role on mechanical signal transduction of the low-magnitude compressive loading. Further understanding of this mechanism may provide insights into the development of new therapies for MSC-based NP regeneration.

## Figures and Tables

**Table 1:** Real-time polymerase chain reaction primers.

Gene	Sequence		Size
Aggrecan	Forward	5′ ATTTCCACACGCTACACCCTG 3′	164 bp
	Reverse	5′ TGGATGGGGTATCTGACTGTC 3′	
Type II	Forward	5′ CAGGATGCCCGAAAATTAGGG 3′	132 bp
Collagen α1	Reverse	5′ ACCACGATCACCTCTGGGT 3′	
Sox-9	Forward	5′ TCAACGGCTCCAGCAAGAACAAG 3′	194 bp
	Reverse	5′ CTCCGCCTCCTCCACGAAGG 3′	
GAPDH	Forward	5′ GGAGTTGCTGTTGAAGTCGCA 3′	532 bp
	Reverse	5′ GGAGTTGCTGTTGAAGTCGCA 3′	

Primers for aggrecan, type II collagen *α*1, Sox-9, and GAPDH were designed from *Mus musculus* gene sequences obtained from the NCBI GenBank and RefSeq databases using Primer 5.0 software.
